# Anxiolytic effects of the flavonoid luteolin in a mouse model of acute colitis

**DOI:** 10.1186/s13041-019-0539-z

**Published:** 2019-12-26

**Authors:** Vinicius M. Gadotti, Gerald W. Zamponi

**Affiliations:** Department of Physiology and Pharmacology. Hotchkiss Brain Institute, Children’s Hospital Research Institute, Cumming School of Medicine, University of Calgary, Calgary, AB Canada

**Keywords:** Colitis, Anxiety, Luteolin, Mood, Dextran sodium sulfate

## Abstract

Anxiety related disorders commonly occur in association with major depressive disorder (MDD) in individuals suffering from peripheral inflammation, with a higher prevalence among IBS patients. We have previously shown that the bioflavonoid luteolin has pronounced analgesic and antidepressant-like effects in mice with dextran sodium sulfate (DSS)-induced colitis. Here, we further evaluate the biological effect of luteolin as a possible anxiolytic agent in DSS treated mice. Anxiolytic action was evaluated using the open field test (OF), the novelty suppressed feeding test (NSFT) and the elevated plus maze test (EPM). Luteolin increased the number of crossings in the center of the OF apparatus, reduced the latency to interact with the food pellet in the NSFT, and increased the time spent in the open arms in the EPM. These results suggest luteolin as a possible natural anxiolytic molecule without sedative effects, thus reinforcing its therapeutic potential for the comorbidities involving peripheral inflammation, pain, mood and anxiety-related disorders.

## Main text

Peripheral inflammation is recognized as an important contributor to anxiety [1] and major depressive disorder [2], with such symptoms observed particularly among patients with inflammatory bowel disease (IBD) [3, 4]. Recent rodent studies using the dextran sodium sulfate (DSS) model demonstrated that the activation of the immune system contributes to the development of anxiety- and depression-related behaviors as consequence of colitis [5–7]. In our previous work, we showed that long-term behavioral abnormalities related to anxiety and depression arise in mice of either sex as a consequence of acute colonic inflammation, in parallel with a marked elevation of interleukin 1beta in the hippocampus, increased infiltration of leukocytes into the brain microvasculature and significant changes of the firing properties of hippocampal pyramidal cells. We further showed that protecting the inflamed gut with the bioflavonoid luteolin (3′,4′,5,7-tetrahydroxyflavone) prevented gut leakage and leukocyte infiltration into the brain, as well as completely reversing pain and depression-related behaviors [7]. The present study extends this work to examine whether luteolin could also represent a potential candidate molecule with anxiolytic properties.

**Fig. 1:**
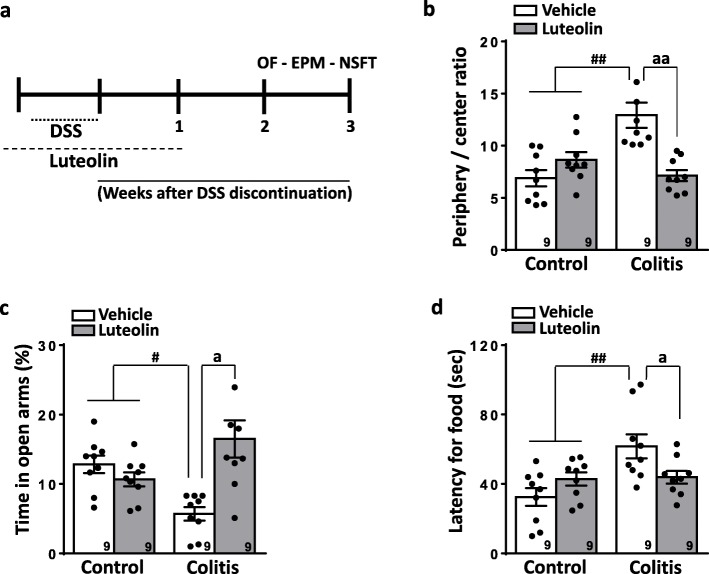
Effect of (**a**) luteolin (15 mg/kg, i.p., once daily for 15 days) in male mice treated with DSS in the (**b**) OF test, (**c**) EPM and (**d**) NSFT test. Each bar represents the mean ± S.E.M. and is representative of 3 independent sets of experiments. Numbers reflect numbers of mice tested. Two-way ANOVA reveals behavioral abnormalities of colitis mice ^#^ *P* < 0.05, ^##^ *P* < 0.01 and luteolin- vs Vehicle-treated mice ^a^ *P* < 0.05, ^aa^ *P* < 0.01

Six week old male mice purchased from Jackson Laboratories received DSS (2.5%, Affymetrix) in their drinking water for 6 consecutive days to induce acute colitis and associated anxious-depressive related behaviors. All behavioral tests were conducted between the 2nd – 3rd weeks following DSS discontinuation, starting from the least to the most stressing testing protocol (Open field (OF) → elevated plus maze (EPM) → novel suppressed feeding test (NSFT)) with a minimum interval of 48 h between trials (Fig. 1a). Experimental procedures were approved by the animal care committee of the University of Calgary. Animal groups were randomized in all experimental trials. Luteolin (15 mg/kg, Tocris) was suspended in dimethylsulfoxide (DMSO, 3%) and diluted in sterile PBS, then administered intraperitoneally (i.p.). Luteolin was delivered once per day for 15 days starting 2 days before beginning of the 6 days DSS treatment protocol. Locomotor activity was assessed and was unaffected by luteolin.

The OF test was used to screen anxiety-related behavior of colitis mice and to analyze the anxiolytic action of luteolin. The OF apparatus consists of a wooden box measuring 40 × 60 × 50 cm with a frontal glass wall to facilitate observation. The floor of the arena is divided into 12 equal squares. Animals were placed in the rear left square and allowed to explore freely for 6 min. The number of crossings in either the center or peripheral quadrants were recorded. Increased exploration in the center quadrants is considered anxiolytic activity. Crossings reflect the number of grid lines that were crossed with all paws [8]. Colitis mice displayed reduced center exploration, indicative of elevated anxiety levels, whereas luteolin increased the number of center crossings, consistent with an anxiolytic effect (Fig. 1b**,** Additional file 1: Figure S1a). Two-way ANOVA revealed significant differences in the periphery/center exploration ratio for colitis and healthy animals [F(3,24) = 12.44; *P* < 0.0001] and for luteolin vs. vehicle treatment interactions[F(8,24) = 1.673; *P* = 0.1567]. No changes in the number of peripheral quadrant crossings were observed (Additional file 1: Figure S1b).

The EPM apparatus consists of two closed and two open arms (all at the dimensions of 6 × 30 cm) elevated 50 cm from the floor. Mice were placed individually in the central part of the maze always facing one of the closed arms and allowed to explore the apparatus for 6 min [9]. The number of entries and the time spent in each arm type (either open or closed) were recorded. Increased exploration in the open arms reflects an anxiolytic effect. In the EPM test, mice subjected to colonic inflammation displayed a significant decrease in the time spent in the open arms whereas colitis mice that received luteolin showed an increase in the time spent in the open arms (Fig. 1c**).** Two-way ANOVA showed differences between colitis and healthy animals [F(3,24) = 7.042; *P* = 0.0015] and for luteolin vs. vehicle treatment interactions [F(7,535) = 8.8; *P* = 0.0016]. No differences were observed among groups regarding the number of entries in either open or closed arms, nor in the total number of entries in all arms **(**Additional file 1: Figure S1c, d, e). The lack of differences in the total number of entries demonstrates that the repeated treatment with luteolin does not alter the locomotor activity of mice. This also reinforces our previous findings showing that there are no differences in the ambulatory behavior of mice treated with luteolin as assessed in the open field test [7]. Altogether, our data indicate that DSS induces anxiety like behaviors that are consistent with previous findings in both male and female mice [5] and which can be attenuated by luteolin treatment.

The NSFT is a conflict paradigm test based on hyponeophagia and is among the most commonly used tests for screening of novel anxiolytic and antidepressant drugs. Following a 24 h food-deprivation period, mice were introduced to a novel 40 × 60 × 50 cm arena (with the floor covered with the same type of type wood shavings as that used in housing cages) and with a single pellet of food placed in the center of the arena. Animals were individually placed in a corner of the arena and the latency of each mouse to interact with the food pellet was recorded. After testing, animals were immediately returned to their home cage. A reduced latency to interact with the food pellet is considered an anxiolytic effect [10]. Here, mice subjected to visceral inflammation exhibited an increased latency to interact with food which was reversed by luteolin (Fig. 1d). Two-way ANOVA revealed significant differences between colitis and healthy mice [F(3,24) = 7.408; *P* = 0.0011] and for luteolin vs. vehicle treatment interactions [F(8,24) = 2.183; *P* = 0.0665].

Luteolin is a naturally occurring flavone that possesses a range of biological actions that include antimicrobial, antiallergic, antidiabetic and antioxidant activities [11]. Luteolin also displays neuroprotective activity linked to the control of the transcription factor nuclear factor kappa-light-chain-enhancer of activated B cells and the production of pro inflammatory cytokines [11, 12]. Particularly in DSS-induced colitis mice, luteolin has been reported to have an anti-inflammatory and protective role in the gut [7, 13, 14]. Our previous findings [7] revealed that luteolin prevents gut leakage and reverses behavioral abnormalities such as pain and depression and here we extend these findings by demonstrating that luteolin is also a molecule with anxiolytic properties. An anxiolytic action of a single systemic luteolin treatment was previously reported in rats subjected to the EPM test [15], and this fits with our data. Therefore, luteolin could potentially be exploited for the development of novel therapeutics for the treatment these co-morbid conditions linking peripheral inflammation to mental disorders.

## Supplementary information

**Additional file 1: Figure S1:** Effect of luteolin (15 mg/kg, i.p., once daily for 15 days) in male mice subjected to colitis on **(a)** the number of center crossings and **(b)** peripheral crossings in the OF test, **(c)** the number of entries in the open arms, **(d)** number of entries in the closed arms, and **(e)** total number of entries in all arms in the EPM. Each bar represents the mean ± S.E.M. and is representative of 3 independent sets of experiments. Numbers reflect numbers of mice tested. Two-way ANOVA reveals behavioral abnormalities of colitis mice ^#^ *P* < 0.05, and luteolin- vs Vehicle-treated mice ^aa^ *P* < 0.01.

## Data Availability

The data used in our study are available from the authors on reasonable request.

## Abbreviations

DSS: Dextran sodium sulfate
EPM: Elevated plus maze
IBD: Inflammatory bowel disease
NSFT: Novelty suppressed feeding test
OF: Open field
