# Supplementary figures and images for: Anxiolytic effects of the flavonoid luteolin in a mouse model of acute colitis
Source: Mol Brain. 2019 Dec 26;12:114. doi: 10.1186/s13041-019-0539-z (PMC6933648; doi:10.1186/s13041-019-0539-z)

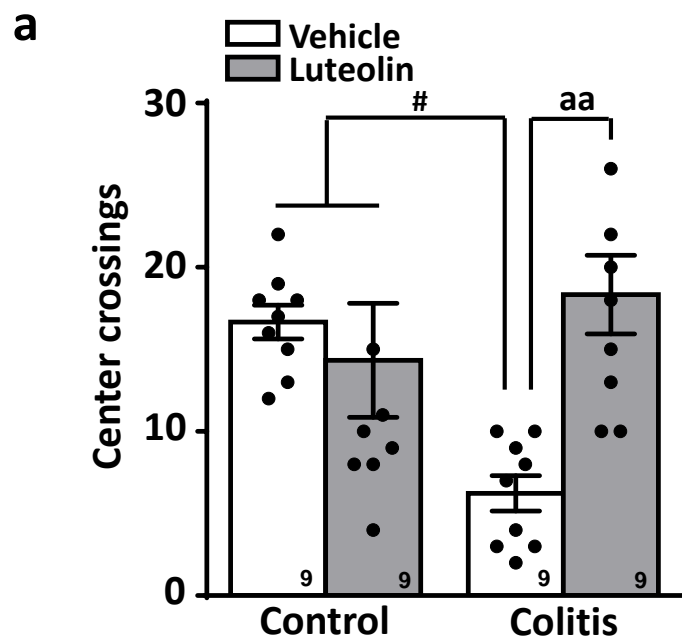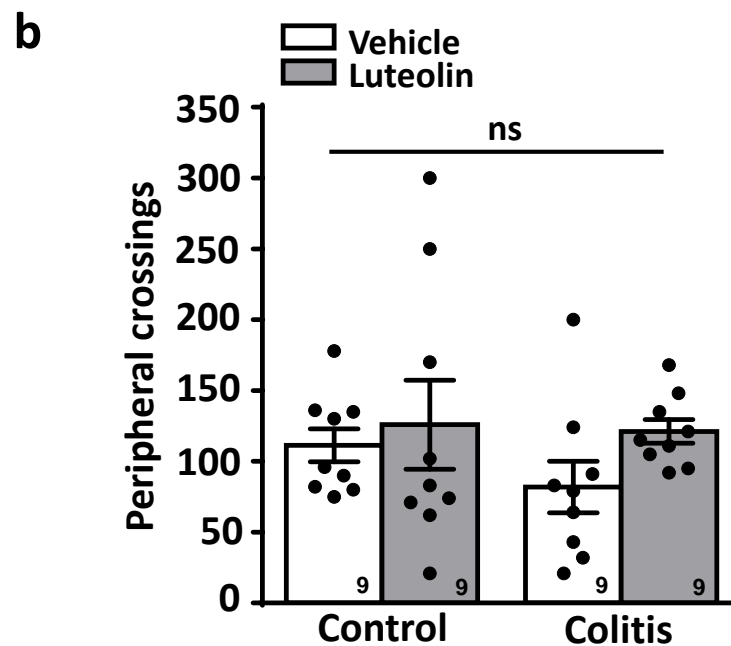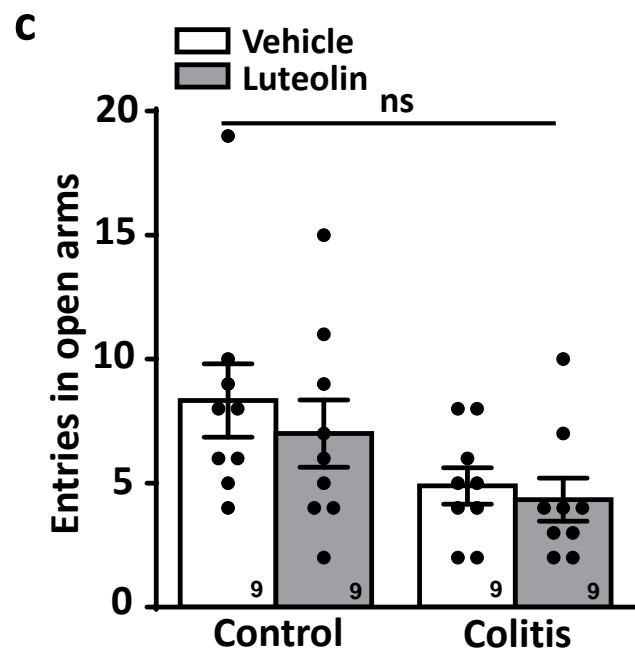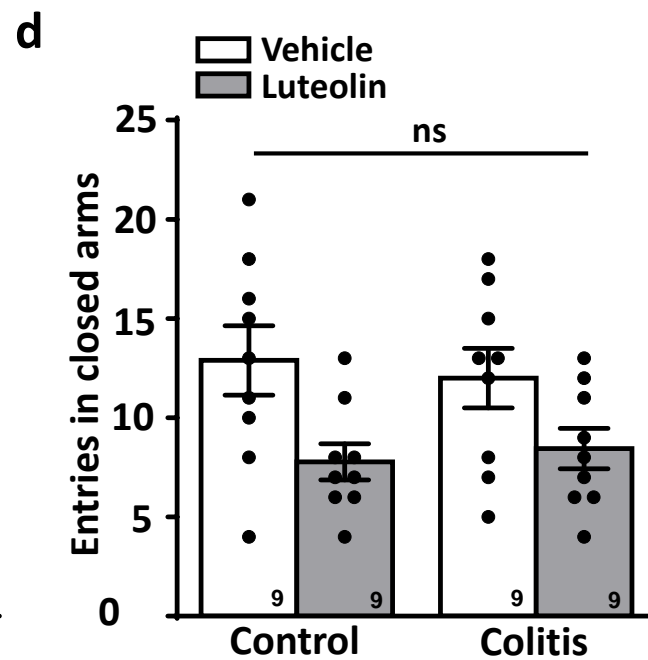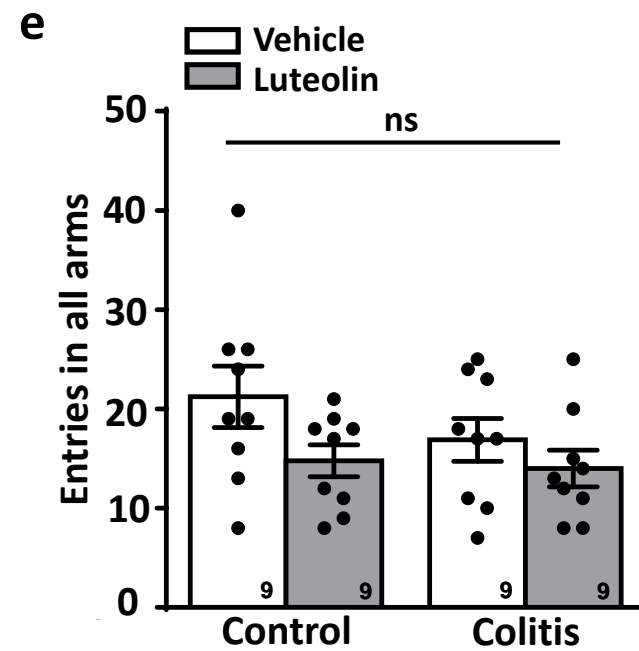

Supplement: Supplementary file 1 — Additional file 1: Figure S1: Effect of luteolin (15 mg/kg, i.p., once daily for 15 days) in male mice subjected to colitis on (a) the number of center crossings and (b) peripheral crossings in the OF test, (c) the number of entries in the open arms, (d) number of entries in the closed arms, and (e) total number of entries in all arms in the EPM. Each bar represents the mean ± S.E.M. and is representative of 3 independent sets of experiments. Numbers reflect numbers of mice tested. Two-way ANOVA reveals behavioral abnormalities of colitis mice # P < 0.05, and luteolin- vs Vehicle-treated mice aa P < 0.01. [file 13041_2019_539_MOESM1_ESM.pdf]
